# Systemic inflammation, multi-organ injury, and acute kidney risk in psittacosis pneumonia: a biomarker-driven clinical characterization

**DOI:** 10.3389/fcimb.2026.1761465

**Published:** 2026-03-09

**Authors:** Yang Song, Na Li, Wei Ni

**Affiliations:** 1 Department of Emergency, Nanjing First Hospital, Nanjing Medical University, Nanjing, China; 2 Department of Endocrinology, Sir Run Run Hospital, Nanjing Medical University, Nanjing, China

**Keywords:** acute kidney injury, blood urea nitrogen to albumin ratio, Chlamydia psittaci, lactate dehydrogenase, pneumonia

## Abstract

**Background:**

Psittacosis pneumonia, which is caused by Chlamydia psittaci, is a systemic inflammatory disease that often results in injury to multiple organs. Although liver and heart involvement are recognized, the incidence, risk factors, and predictors for acute kidney complications are not well known.

**Methods:**

Our retrospective cohort study included 123 patients, comprising 47 individuals diagnosed with psittacosis pneumonia and 76 individuals with typical community-acquired pneumonia (CAP). These patients were admitted to Nanjing First Hospital, affiliated with Nanjing Medical University, between June 2019 and July 2024. The study conducted an analysis of clinical profiles, laboratory markers, and patient outcomes. The predictive efficacy of the Blood Urea Nitrogen to Albumin Ratio (BAR) and Lactate Dehydrogenase (LDH) in forecasting acute kidney injury (AKI) was assessed through the application of receiver operating characteristic (ROC) curve analysis.

**Results:**

In comparison to typical CAP, psittacosis pneumonia is characterized by markedly elevated systemic inflammation, as evidenced by increased levels of procalcitonin (PCT), C-reactive protein (CRP), and interleukin-6 (IL-6). Additionally, it presents with more severe lymphocytopenia, hypoalbuminemia, and extensive multi-organ damage, with a pronounced impact on hepatic and myocardial tissues. The incidence of AKI was significantly greater in the psittacosis group compared to the control group (34.0% versus 12.1%, *P=*0.003). In the psittacosis cohort, AKI demonstrated an independent association with increased levels of LDH (*P* = 0.01) and the BAR (*P* < 0.001), whereas no such association was observed with traditional inflammatory markers. The BAR (AUC = 0.88) and LDH (AUC = 0.79) demonstrated effective predictive capabilities for AKI, with their combined application enhancing sensitivity to 85.71% and specificity to 87.50%. The implementation of targeted therapy using omadacycline resulted in prompt clinical and biochemical improvements across all patients.

**Conclusion:**

Psittacosis pneumonia constitutes a distinct systemic immuno-inflammatory syndrome that presents a significant risk for AKI. The composite biomarker BAR and the cellular injury marker LDH demonstrate superior predictive capabilities for AKI compared to traditional inflammatory indices, thereby providing accessible tools for early risk stratification. These findings emphasize the necessity of recognizing psittacosis as a unique clinical entity and advocate for vigilant monitoring of renal function in affected patients.

## 1 Introduction

Psittacosis, a zoonotic infectious disease caused by the bacterium Chlamydia psittaci, is predominantly transmitted to humans through the inhalation of aerosols originating from infected avian species or poultry. This mode of transmission often results in the manifestation of psittacosis as an atypical form of community-acquired pneumonia (CAP) (Hogerwerf et al., 2017; Sheng et al., 2025). In clinical settings, psittacosis presents as a systemic inflammatory syndrome. Although the lungs are the primary target organ, frequently manifesting as pneumonia that is initially indistinguishable from conventional bacterial CAP, the pathogen’s capacity for hematogenous dissemination results in multi-organ involvement (Knittler and Sachse, 2015; Radomski et al., 2016). The clinical presentation is notably diverse, encompassing a range from mild, influenza-like symptoms to severe pneumonia, which may be further complicated by acute respiratory distress syndrome and multiple organ dysfunction syndrome (Branley et al., 2014; Yang et al., 2022).

Severe psittacosis is marked by its inclination to injure organs beyond the lungs. Liver damage, shown by high transaminase levels, is a recognized and common extrapulmonary effect (Fang et al., 2025; Guo et al., 2023). Evidence of muscular damage, particularly rhabdomyolysis as demonstrated by significant elevations in creatine kinase (CK), is commonly reported. Elevated lactate dehydrogenase (LDH) further indicates systemic cellular injury, further emphasizing the infection’s systemic and aggressive nature (Li et al., 2022; Liu et al., 2024). In comparison to hepatic and myocardial damage, acute kidney injury (AKI) in psittacosis has been insufficiently studied, despite its critical role in determining mortality and morbidity in severe systemic infections (Hoste et al., 2018; Mehta et al., 2007). The severe inflammatory response and possible direct cellular damage in psittacosis might lead to a unique form of kidney damage, with its risk factors still not well understood (Kellum and Prowle, 2018; Zhang et al., 2022).

The early and precise prediction of AKI is important for ensuring timely supportive care and avoiding negative outcomes. While traditional inflammatory markers such as C-reactive protein (CRP) and procalcitonin (PCT) indicate systemic inflammation, they might not specifically predict kidney complications in this particular pathophysiology (Ostermann et al., 2020). Therefore, it is crucial to investigate new composite biomarkers that combine various pathological pathways. The Blood Urea Nitrogen to Albumin Ratio (BAR) serves as an accessible indicator that simultaneously reflects renal perfusion stress, as indicated by blood urea nitrogen levels, and the severity of systemic inflammation or nutritional catabolism, as indicated by albumin levels. This metric has demonstrated significant prognostic potential in a range of critical illnesses (Huang et al., 2020; Yang et al., 2025). Likewise, LDH, which indicates overall cellular damage and tissue hypoxia, could reflect the level of systemic injury (Henry et al., 2020). Research has not explored the predictive value of BAR and LDH for AKI in psittacosis pneumonia.

Moreover, the literature lacks a thorough, direct comparison of the clinical phenotypes of psittacosis pneumonia and typical CAP, specifically addressing detailed inflammatory, immunological, nutritional, and multi-organ injury profiles. This delineation is essential for recognizing psittacosis as a distinct clinical entity with a unique disease burden, frequently marked by a more rapid clinical deterioration and an extended hospital stay (Tang et al., 2023).

Consequently, the objectives of this study were formulated as follows: (1) to conduct a comparative analysis of the clinical, laboratory, and inflammatory profiles of patients diagnosed with psittacosis pneumonia versus those with conventional CAP; (2) to characterize the pattern of multi-organ injury in psittacosis, with a specific emphasis on elucidating the incidence, severity, and associated risk factors for AKI; (3) to evaluate and compare the predictive performance of novel biomarkers, specifically BAR and LDH, for the development of AKI in psittacosis pneumonia; and (4) to describe the clinical and biochemical response to targeted anti-Chlamydia therapy with omadacycline.

## 2 Materials and methods

### 2.1 Study design and subjects

This retrospective cohort study involved 123 patients (47 with psittacosis pneumonia and 76 with typical CAP) and was conducted at the department of Emergency and department of Respiratory of Nanjing First Hospital Affiliated to Nanjing Medical University. We consecutively enrolled adult patients (aged≥18 years) hospitalized from June 2019 to July 2024 with a confirmed diagnosis of either psittacosis pneumonia or typical CAP. This study was approved by the Ethics Committee of Nanjing First Hospital Affiliated to Nanjing Medical University, and all clinical test indicators were agreed upon by the patients or their immediate family members (Ethics Approval Number: KY20201102-03). The diagnosis of psittacosis pneumonia was confirmed by positive metagenomic next-generation sequencing (mNGS) testing for Chlamydia psittaci nucleic acid in bronchoalveolar lavage fluid or sputum samples (Li et al., 2022). The typical CAP group consisted of patients diagnosed with bacterial pneumonia based on clinical presentation, radiological findings, and in accordance with the Infectious Diseases Society of America and American Thoracic Society (IDSA/ATS) consensus guidelines for CAP (Jackson et al., 2020). All patients in the typical CAP group had positive conventional microbiological cultures (e.g., sputum, blood) for common bacterial pathogens, but with negative mNGS results for Chlamydia psittaci and other atypical pathogens. Patients with end-stage renal disease, chronic kidney disease stage 4 or 5 (baseline estimated glomerular filtration rate <30 mL/min/1.73 m²), active malignancy, or incomplete medical records were excluded from the analysis.

### 2.2 Laboratory assessments

mNGS Detection Method: Pathogen detection was performed using commercial mNGS services. Briefly, nucleic acids were extracted from clinical samples, followed by library construction and high-throughput sequencing. Sequencing data were analyzed by aligning reads to comprehensive pathogen databases for microbial identification (Li et al., 2022).

Parameters and Abnormalities: Upon admission, demographic data, clinical symptoms, and history of bird/poultry exposure were recorded. Laboratory parameters were measured using standard automated clinical analyzers in the hospital central laboratory. Key assessments included: 1) Inflammatory markers: Serum PCT and CRP were measured by immunoturbidimetric assay; IL-6 was quantified by chemiluminescence immunoassay. 2) Immune cells: Complete blood count with differential was performed using automated hematology analyzers. 3) Nutritional status: Albumin was measured by bromocresol green method. 4) Organ injury markers: Hepatic enzymes [alanine aminotransferase (ALT), aspartate aminotransferase (AST), and gamma-glutamyl transferase (γ-GT)], myocardial enzymes [CK, creatine kinase-MB (CK-MB), LDH], and renal function markers [blood urea nitrogen (BUN), serum creatinine (Scr)] were all determined using enzymatic methods on biochemical analyzers. 5) Derived indices: The Blood Urea Nitrogen to Albumin Ratio (BAR = BUN [mmol/L]/Albumin [g/L]) was calculated, a composite marker reflecting renal perfusion stress and systemic inflammation/nutritional status (Shi et al., 2024). The estimated glomerular filtration rate (eGFR) was calculated using the CKD-EPI formula. Abnormalities were defined according to the hospital’s reference ranges.

### 2.3 Treatment protocol and monitoring

Upon etiological confirmation of Chlamydia psittaci infection, targeted antimicrobial therapy with intravenous omadacycline was initiated. The regimen consisted of a 200 mg loading dose on day 1, followed by a maintenance dose of 100 mg administered intravenously every 24 hours (infused over 60 minutes) for a total duration of 7 to 10 days, guided by clinical and biochemical response. The infusion was administered over 60 minutes. Dosage adjustments were not required for patients with renal impairment, in accordance with the drug’s prescribing information.

### 2.4 Efficacy assessment

The efficacy of omadacycline therapy was assessed at day 7–10 after treatment initiation using pre-specified composite criteria (File et al., 2025; Stets et al., 2019). Treatment outcomes were classified into three categories:

Markedly effective: Complete defervescence (body temperature < 37.3 °C sustained for ≥ 48 hours), resolution of clinical symptoms (cough, dyspnea), and ≥75% reduction in baseline C-reactive protein (CRP) level, accompanied by partial or complete resolution of pulmonary infiltrates on chest imaging.

Effective: Defervescence achieved, significant improvement in clinical symptoms, and 50–75% reduction in baseline CRP level, with stable or improved chest imaging.

Ineffective: Persistent fever or clinical deterioration requiring escalation of antimicrobial therapy or intensive care unit (ICU) transfer.

The total efficacy rate was calculated as the proportion of patients classified as either markedly effective or effective among all patients who received omadacycline therapy.

### 2.5 Diagnostic criteria

AKI was diagnosed and staged according to the Kidney Disease: Improving Global Outcomes (KDIGO) 2012 clinical practice guidelines (Khwaja, 2012). AKI was defined as meeting any one of the following criteria within a 48-hour period: 1) An increase in Scr by ≥0.3 mg/dL (≥26.5 μmol/L); 2) An increase in Scr to ≥1.5 times the baseline value, which is known or presumed to have occurred within the prior 7 days; 3) Urine volume <0.5 mL/kg/h for 6 hours. The baseline Scr was defined as the most recent outpatient value within 3 months before admission or the first Scr upon admission if no prior value was available.

### 2.6 Statistical analysis

Statistical analyses were performed using SPSS software (version 26.0). Continuous variables were presented as mean[standard deviation (SD)] based on their distribution, assessed by the Shapiro-Wilk test. Comparisons between two groups were made using the Student’s t-test [mean (SD)] or the Mann-Whitney U test [median (IQR)], as appropriate. Categorical variables were expressed as numbers (percentages) and compared using the Chi-square test or Fisher’s exact test. Univariate analyses were conducted to identify factors associated with AKI in the psittacosis cohort. The predictive performance of BAR and LDH for AKI was evaluated using ROC curve analysis, with the optimal cut-off value determined by the Youden index. The area under the ROC curve (AUC) was reported with its 95% confidence interval (CI). A two-sided P-value <0.05 was considered statistically significant.

## 3 Result

### 3.1 Comparative clinical profiles of psittacosis pneumonia and typical pneumonia: significant differences in inflammatory, immune, and nutritional indicators

This study included 123 patients, comprising 47 diagnosed with Psittacosis pneumonia and 76 with typical pneumonia. As shown in **Table 1**, there were no significant differences in age or sex between the two groups (*P*>0.05). While a well-documented history of contact with birds or poultry is a recognized characteristic of psittacosis, not all patients within the psittacosis cohort reported such exposure (n=20, 41.67%). In contrast, no individuals in the common pneumonia group reported this history (*P* < 0.001).

**Table 1:** Comparison of baseline characteristics and clinical indicators between patients with *C. psittaci* pneumonia and typical pneumonia.

Variables	Typical Pneumonia(n=76)	C. psittaci Pneumonia(n=47)	*P*-value
Gender(M/F)	35/41	24/23	0.59
Age (years)	58.83 (20.65)	61.15 (13.16)	0.71
Contact with birds or poultry	0 (0%)	20 (41.67%)	<0.001
Inflammatory variables			
PCT (ng/mL)	0.07 (0.03-0.17)	0.57 (0.17-1.54)	<0.001
CRP (mg/L)	30.54 (9.70-59.41)	132.34 (80.5-185.61.)	<0.001
IL-6 (pg/mL)	6.60 (2.26-24.18)	33.81 (6.73-133.32)	<0.001
Immune variables			
WBC (×10^9^/L)	9.56 (10.64)	7.94 (2.39)	0.31
Neutrophil count (×10^9^/L)	5.64 (6.00)	6.09 (2.59)	0.63
Percentage of Neutrophilic granulocyt (%)	70.84 (14.89)	82.67 (7.91)	<0.001
Monocyte count (×10^9^/L)	0.60 (0.76)	0.53 (0.89)	0.67
Percentage of monocytes (%)	7.08 (3.02)	6.07 (3.24)	0.10
Lymphocyte count (×10^9^/L)	2.31 (2.80)	0.72 (0.40)	<0.001
Percentage of lymphocytes (%)	22.50 (11.19)	15.95 (11.98)	0.003
Nutrition and reserve variables			
Albumin (g/L)	38.05 (35.33-40.10)	31.70 (28.50-34.58)	<0.001
Hb (g/L)	125.74 (22.75)	121.91 (15.75)	0.15

PCT, procalcitonin; CRP, C-reactive protein; IL-6, interleukin-6; WBC, white blood cell count; Hb, hemoglobin. PCT (ng/mL, 0-0.1); CRP (mg/L, <10); IL-6 (pg/mL, 0-7); Percentage of Neutrophilic granulocyt (%, 40-75); Lymphocyte count(×10^9^/L, 20-50); Percentage of lymphocytes (%, 20-50); Albumin (g/L, 40-55). Data are n (%), mean (SD), and median (IQR). Comparisons were made using the Student’s t-test or Mann-Whitney U test, as appropriate. P < 0.05 was considered statistically significant.

In relation to inflammatory markers, the levels of PCT, CRP, and IL-6 were markedly elevated in the psittacosis pneumonia cohort compared to the common pneumonia cohort, with all differences reaching statistical significance (*P* < 0.001). Concerning immune indicators, there were no statistically significant differences observed in white blood cell count, neutrophil count, monocyte count, or monocyte percentage. Nevertheless, the psittacosis group demonstrated a significantly higher neutrophil percentage (*P* < 0.001) and significantly lower lymphocyte count and percentage (*P* < 0.001 and *P=*0.003, respectively). Regarding nutritional and reserve parameters, albumin levels were significantly reduced in the psittacosis pneumonia group (*P* < 0.001), whereas hemoglobin (Hb) levels did not show a statistically significant difference between the groups. The results indicate that psittacosis pneumonia differs significantly from typical CAP in its clinical presentation, warranting its consideration as a separate clinical entity.

### 3.2 The clinical profile of psittacosis pneumonia: a pattern characterized by multi-organ involvement

In addition to the distinct inflammatory, immune, and nutritional profiles observed between the two groups, we further evaluated the impact on multiorgan function. As illustrated in **Table 2**, patients with psittacosis pneumonia exhibited significantly elevated markers of liver injury, myocardial involvement, and, to a lesser extent, renal impairment. Specifically, hepatic involvement was evidenced by significantly higher levels of ALT, AST, and γ-GT in the psittacosis pneumonia group (*P* < 0.001). Regarding markers of muscular and generalized cellular injury, CK and LDH levels were markedly elevated in the psittacosis group (both *P* < 0.001), whereas CK-MB levels did not differ significantly between the groups. The pronounced rise in total CK suggests rhabdomyolysis, while elevated LDH indicates systemic cellular injury or hypoxia. In terms of renal parameters, the Scr level was elevated in the psittacosis group (*P* = 0.04), and the clinical incidence of AKI was significantly higher compared to the common pneumonia group (34.0% *vs*. 12.1%, *P* = 0.003). Although BUN and eGFR levels did not reach statistical significance, both exhibited trends suggestive of impaired renal function in the psittacosis pneumonia group.

**Table 2:** Comparison of organ injury-related biochemical markers between patients with *C. psittaci* pneumonia and typical pneumonia.

Damage criterion	Related variables	Typical Pneumonia(n=76)	C. psittaci Pneumonia(n=47)	*P*-value
Liver	ALT (U/L)	18.50 (13.00-28.50)	62.00 (32.00-114.00)	<0.001
	AST (U/L)	21.50 (15.00-28.78)	87.00 (35.00-172.00)	<0.001
	r-GT (U/L)	21.00 (16.00-46.00)	56.50 (29.10-129.50)	<0.001
Markers of Tissue Injury	CK (U/L)	64.00 (40.00-107.00)	198.00(78.75-878.75)	<0.001
	CK-MB (U/L)	11.90 (8.00-16.00)	12.00 (7.00-20.10)	0.57
	LDH (IU/L)	186.50 (164.75-237.75)	282.30 (205.00-509.00)	<0.001
kidney	Scr (umol/L)	63.75 (54.30-75.59)	74.52 (57.10-99.67)	0.04
	BUN (mmol/L)	4.63 (3.51-5.70)	4.79 (3.66-6.23)	0.09
	eGFR	95.58 (21.71)	84.48 (27.37)	0.06

ALT, alanine aminotransferase; AST, aspartate aminotransferase; r-GT, gamma−glutamyl transferase; CK, creatine kinase; CK-MB, creatine kinase-MB; LDH, lactate dehydrogenase; Scr, serum creatinine; BUN, blood urea nitrogen; eGFR, estimated glomerular filtration rate. ALT (U/L, 7-40); AST (U/L, 13-45); r-GT (U/L, 7-45); CK (U/L, 40-200);.

LDH (IU/L, 120-250); Scr (umol/L, 57-111); BUN (mmol/L, 3.6-9.5).Normally distributed variables are presented as mean (SD), and comparisons were performed using the Student’s t-test. Non-normally distributed variables are presented as median (interquartile range, IQR), and comparisons were performed using the Mann–Whitney U test. *P* < 0.05 was considered statistically significant.

### 3.3 Psittacosis pneumonia is characterized by more rapid onset to admission and prolonged hospitalization

In addition to variations in clinical and organ injury markers, the two cohorts exhibited significant differences in disease trajectory and healthcare utilization. **Table 3** provides a comparative analysis of the temporal indicators between the groups. The interval from symptom onset to hospital admission was significantly shorter in the psittacosis pneumonia cohort compared to the common pneumonia cohort [5.0 (3.0-7.0) days *vs*. 7.0 (4.0-8.8) days, *P* < 0.001]. In contrast, the duration of hospitalization was notably longer in the psittacosis pneumonia group [11.0 (8.0-13.0)days *vs*. 6.5 (5.0-8.0) days, *P* < 0.001]. These findings suggest that psittacosis pneumonia is characterized by a distinct clinical timeline, with a significantly expedited admission process but an extended hospital stay, underscoring its unique disease progression and increased healthcare demands.

**Table 3:** Comparison of time to admission and length of hospital stay between patients with *C. psittaci* pneumonia and typical pneumonia.

Variables	Typical Pneumonia(n=76)	C. psittaci Pneumonia(n=47)	*P*-value
The time from the onset of the disease to admission (d)	7.0 (4.0-8.8)	5.0 (3.0-7.0)	<0.001
Prolonged hospitalization (d)	6.5 (5.0-8.0)	11.0 (8.0-13.0)	<0.001

Data are presented as median (IQR). P < 0.05 was considered statistically significant.

### 3.4 Risk factors for acute kidney injury in psittacosis pneumonia: significant associations with LDH, BAR, and prolonged hospitalization

While hepatic and myocardial injury in psittacosis pneumonia have been well documented, the occurrence and correlates of AKI in this population are less clearly defined.

**Figure 1:**
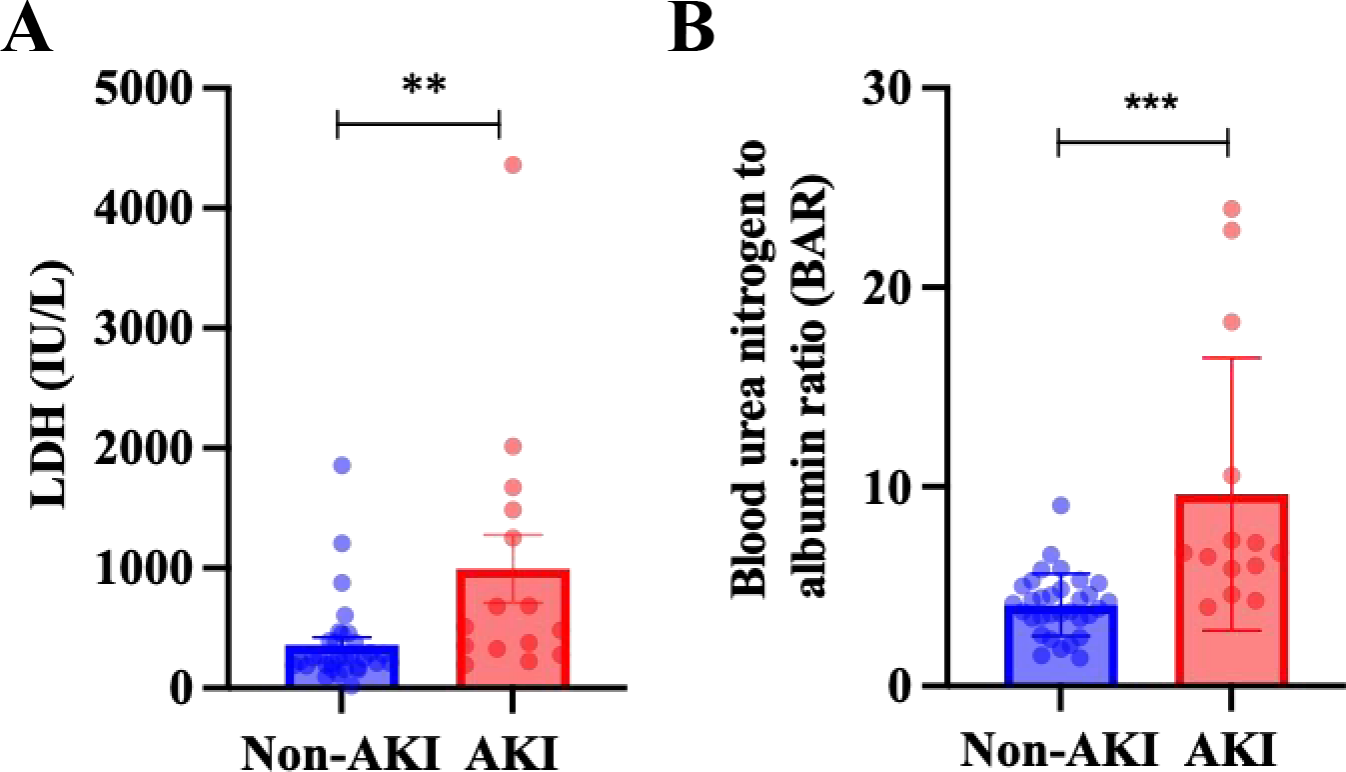
Comparison of LDH or BAR levels between two groups. **(A)**LDH; **(B)** BAR.

The preceding analysis suggests that AKI is a prevalent complication associated with psittacosis pneumonia. To further elucidate its characteristics and identify potential risk factors, we performed a subgroup analysis on the 47 patients diagnosed with psittacosis pneumonia, categorizing them based on the presence of AKI. Among these patients, 16 (34.0%) developed AKI. As presented in **Table 4**, there were no statistically significant differences between the AKI and non-AKI groups in terms of baseline characteristics, including baseline renal function (*P* = 0.40). Critically, the Peak Scr was significantly higher in the AKI group (*P* < 0.001), confirming the acute deterioration of renal function. There were no statistically significant differences between the AKI and non-AKI groups in terms of baseline characteristics (such as sex and age), systemic inflammatory markers (including PCT, CRP, and IL-6), nutritional status indicators (albumin and Hb), liver injury markers (ALT, AST, and r-GT), or CK levels. However, the AKI group exhibited the following distinct features: significantly higher LDH levels [509.00 (325.0-1486.0) IU/L *vs*. 253.50 (185.75-392.75) IU/L, *P* = 0.01, **Figure 1A**], a markedly elevated BAR level [6.68 (5.57-12.48) *vs*. 4.01 (3.40-4.98), *P* < 0.001, **Figure 1B****]**, and a significantly prolonged hospital stay [13.8 (4.9) days *vs*. 9.6 (2.9) days, *P* = 0.001].

**Table 4:** Comparison of clinical characteristics between psittacosis pneumonia patients with and without AKI. .

Variables	Non-AKI(n=31)	AKI(n=16)	*P*-value
Gender(M/F)	14/17	9/7	0.52
Age (years)	61.93 (10.42)	67.40 (8.70)	0.09
Neutrophil count (×10^9^/L)	5.49 (2.18)	5.76 (2.93)	0.10
Percentage of Neutrophilic granulocyt (%)	83.65 (77.23-86.73)	88.1 (84.3-91.5)	0.26
Monocyte count (×10^9^/L)	0.44 (0.25)	0.56 (1.6)	0.47
Percentage of monocytes (%)	4.9 (3.29)	4.16 (2.24)	0.50
Lymphocyte count (×10^9^/L)	0.84 (0.40)	0.94 (0.21)	0.46
Percentage of lymphocytes (%)	19.03 (12.57)	17.38 (7.33)	0.33
Hb (g/L)	120.53 (15.53)	125.07 (16.38)	0.39
PCT (ng/mL)	1.29 (0.1-3.68)	1.93 (0.80-4.82)	0.10
CRP (mg/L)	131.00 (100.55-163.44)	150.00 (131.79-230.78.)	0.23
Albumin	33.30 (29.80-34.86)	29.45 (26.40-32.05)	0.09
ALT (U/L)	82.80 (80.71)	86.23 (91.74)	0.27
AST (U/L)	86.35 (32.00-169.75)	79.50 (62.00-137.00)	0.08
r-GT (U/L)	55.00 (27.00-128.00)	94.5 (32.95-171.25)	0.07
CK (U/L)	675.64 (205.6)	1058.91 (908.59)	0.49
LDH (IU/L)	253.50 (185.75-392.75)	509.00 (325.0-1486.0)	0.01
IL-6 (pg/mL)	35.26 (8.14-107.33)	40.85 (18.92-123.02)	0.11
Baseline Scr (umol/L)	62.19 (11.84)	66.34 (17.59)	0.40
Peak Scr (umol/L)	63.08 (12.98)	151.77 (118.39)	<0.001
BAR	4.01 (3.40-4.98)	6.68 (5.57-12.48)	<0.001
Prolonged hospitalization (d)	9.6 (2.9)	13.8 (4.9)	0.001

AKI, acute kidney injury; PCT, procalcitonin; CRP, C−reactive protein; IL−6, interleukin−6; ALT, alanine aminotransferase; AST, aspartate aminotransferase; r−GT, gamma−glutamyl transferase; CK, creatine kinase; LDH, lactate dehydrogenase; BAR, Blood Urea Nitrogen to Albumin Ratio. Data are mean (SD) and median (IQR). Group comparisons were made using the Student’s t−test or Mann–Whitney U test for continuous variables and the Chi−square or Fisher’s exact test for categorical variables. P < 0.05 was considered statistically significant.

In conclusion, the present subgroup analysis demonstrates a notably elevated incidence of AKI among patients diagnosed with psittacosis pneumonia. The onset of AKI did not correlate with conventional inflammatory markers or isolated organ injury indices. However, it showed a significant association with increased LDH levels, an elevated BAR level, and extended durations of hospitalization. These findings imply that AKI may function as a critical integrative marker of disease severity in psittacosis pneumonia. The underlying mechanisms may involve systemic tissue hypoxia or injury, as suggested by elevated LDH levels, and nutritional or metabolic imbalances, as indicated by the BAR, rather than being solely attributable to direct renal infection or traditional inflammatory pathways.

### 3.5 Predictive value of BAR and LDH for acute kidney injury in psittacosis pneumonia and the advantage of their combined use

**Figure 2:**
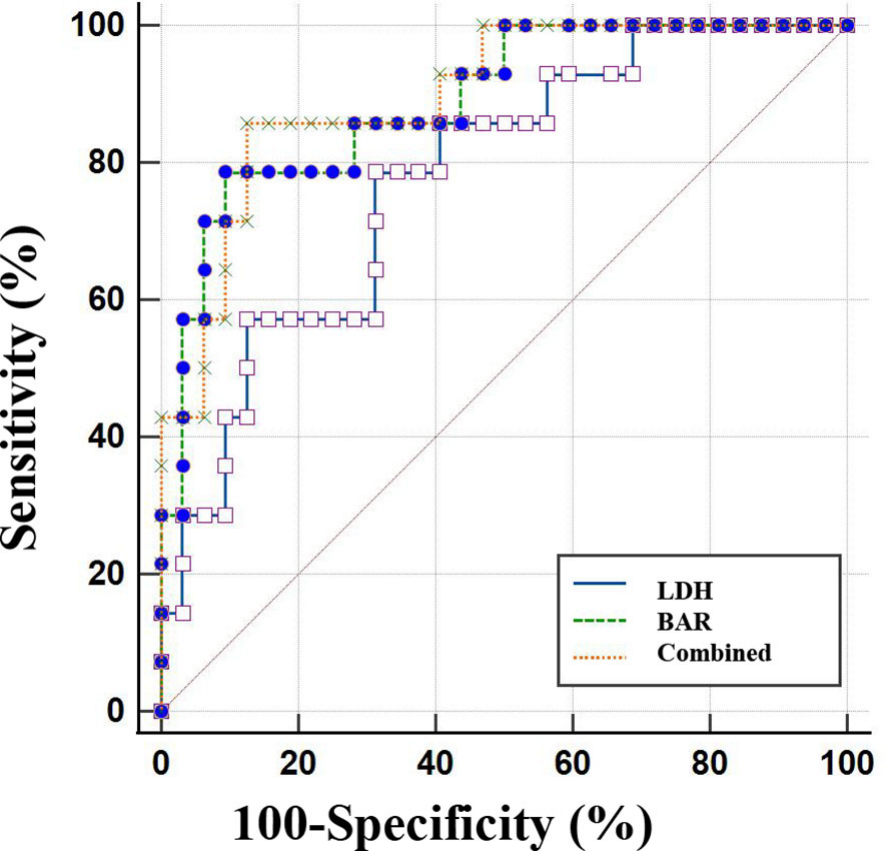
ROC curve analysis of BAR, LDH, and their combination for predicting AKI in psittacosis pneumonia.

Through the analysis of the ROC curve, the optimal cutoff values for predicting AKI in cases of psittacosis pneumonia were identified. For LDH level, a threshold of 294 IU/L was established, resulting in a sensitivity of 80% and a specificity of 68.75%, with the AUC of 0.79 (95% CI: 0.648–0.896, *P* < 0.001). In terms of the BAR level, the optimal cutoff was determined to be 5.83, which provided a sensitivity of 78.57% and a specificity of 90.62%, with an AUC of 0.88 (95% CI: 0.761–0.962, *P* < 0.001). Importantly, the combination of BAR and LDH exhibited enhanced diagnostic performance, achieving a sensitivity of 85.71% and a specificity of 87.50%, thereby surpassing the efficacy of either biomarker when used independently (**Figure 2**, **Table 5**).

**Table 5:** BAR, LDH and a combination of the two for prediction of AKI in psittacosis pneumonia.

Variables	AUC	Sensitivity	Specificity
BAR	0.88	78.57	90.62
LDH (IU/L)	0.79	80.00	68.75
Combination	0.90	85.71	87.50

BAR, Blood Urea Nitrogen to Albumin Ratio; LDH, lactate dehydrogenase; AUC, area under the receiver operating characteristic curve.

### 3.6 Targeted therapy with omadacycline significantly improves clinical outcomes in psittacosis pneumonia

Before the identification of the pathogen, empirical antibiotic therapy was administered to 25 patients: 13 received β-lactams alone, 9 were treated with a combination of β-lactams and quinolones, 2 received β-lactams plus macrolides, and 1 was administered quinolones alone. Upon confirmation of Chlamydia psittaci infection, all patients were transitioned to omadacycline.

Early clinical and biochemical responses were assessed at day 3–5 after omadacycline initiation.

Within this period, 24 patients (96.0%) exhibited normalization of body temperature, and significant reductions were observed in key inflammatory markers (neutrophil percentage, CRP, and PCT) as well as in biochemical indicators of hepatic, myocardial, and renal injury (ALT, AST, LDH, CK, and Scr)(**Tables 6**, **7**; all *P* < 0.05).

**Table 6:** Early changes in body temperature and inflammatory markers at day 3–5 after omadacycline therapy in patients with psittacosis pneumonia.

Parameter comparison	Pre-treatment	Post-treatment (Day 3-5)	*P-*value
Temperature (°C)	39.40 (0.57)	36.81 (0.25)	<0.001
PCT (ng/L)	1.87 (0.70-3.72)	0.37 (0.06-0.72)	0.003
CRP (mg/L)	142.07 (128.50-244.10)	35.97 (19.05-56.76)	<0.001
N%	84.54 (4.46)	66.75 (10.89)	<0.001

PCT, procalcitonin; CRP, C−reactive protein; N%, neutrophil percentage.Normally distributed paired differences were analyzed using the paired t-test and presented as mean (SD); non-normally distributed paired differences were analyzed using the Wilcoxon signed-rank test and presented as median (IQR). *P* < 0.05 was considered statistically significant.

**Table 7:** Early changes in biochemical parameters of organ injury at day 3–5 after omadacycline therapy in psittacosis pneumonia.

Paramete comparison	Pre-treatment	Post-treatment (Day 3–5)	*P-*value
ALT (U/L)	137.20 (93.53)	71.94 (78.20)	0.02
AST (U/L)	163.28 (58.10-321.20)	73.24 (30.04-117.82)	<0.001
LDH (U/L)	534.21 (353.21-1501.03)	264.17 (173.45-488.63)	<0.001
CK (U/L)	997.00 (314.18-1673.37)	308.47 (72.13-993.42)	0.006
Scr (umol/L)	100.36 (89.69)	89.73 (75.90)	0.03

ALT, alanine aminotransferase; AST, aspartate aminotransferase; LDH, lactate dehydrogenase; CK, creatine kinase; Scr, serum creatinine. Normally distributed paired differences were analyzed using the paired t-test and presented as mean (SD); non-normally distributed paired differences were analyzed using the Wilcoxon signed-rank test and presented as median (IQR). *P* < 0.05 was considered statistically significant.

Radiological assessment demonstrated resolution of pulmonary lesions in all 24 patients who underwent follow-up chest CT approximately one week after treatment initiation. In four patients, transient radiographic progression was observed, which is likely indicative of a delay in imaging improvement relative to clinical recovery.

Final treatment efficacy was evaluated at day 7–10 using the pre-specified composite criteria. Among the 25 patients treated with omadacycline, 14 were classified as markedly effective and 10 as effective, while 1 patient was considered ineffective due to persistent fever requiring ICU transfer. This yielded a total efficacy rate of 96.15% (24/25). Omadacycline was well-tolerated, with no significant adverse events reported throughout the treatment period.

## 4 Discussion

This study provides a comprehensive comparative analysis of the clinical profiles and outcomes between patients with psittacosis pneumonia and those with typical CAP. Our findings corroborate and expand upon previous observations by identifying a distinct clinical syndrome marked by significant lymphocytopenia, severe systemic inflammation, and multi-organ injury. The high incidence of AKI, its unique association with composite biomarkers such as the Biomarker for the Blood Urea Nitrogen to Albumin Ratio (BAR) and LDH rather than conventional inflammatory markers, and the distinct disease progression emphasize the importance of recognizing psittacosis as a separate clinical entity with specific management considerations.

Initially, our data underscore that psittacosis should be understood as a fundamentally systemic inflammatory disease rather than solely a pulmonary infection. The distinctive triad of significant lymphocytopenia, hyperinflammation (evidenced by elevated levels of CRP, PCT, and IL-6), and hypoalbuminemia differentiates it from typical CAP, where such pronounced immune dysregulation is less prevalent (Fang et al., 2025; Yang et al., 2022). This pattern is consistent with the broader paradigm of sepsis-induced immunosuppression, wherein an initial hyperinflammatory response may be succeeded by or coexist with a state of functional immune paralysis, characterized by lymphocyte depletion and anergy (Boomer et al., 2014; Hotchkiss et al., 2013). The marked reduction in lymphocyte count observed in our psittacosis cohort may therefore represent more than a mere indicator of severity; it could signify a state of acquired immunosuppression that potentially impairs pathogen clearance and contributes to disease progression and organ damage (Boomer et al., 2014). This immunological context may facilitate the systemic dissemination of Chlamydia psittaci, thereby accounting for the high incidence of extra-pulmonary involvement.

Importantly, acute kidney injury (AKI) emerged as a common and severe complication. This is consistent with the observed systemic injury pattern—predominant liver damage and rhabdomyolysis (marked by high CK but normal CK-MB)—which aligns with the pathogen’s hematogenous dissemination (Guo et al., 2023; Liu et al., 2024). In our cohort, the dramatic increase in total CK coupled with normal CK-MB levels strongly suggests that skeletal muscle (rhabdomyolysis), rather than cardiac muscle, is a major site of injury in severe psittacosis, which may also contribute to the risk of AKI through mechanisms such as myoglobinuria. Importantly, AKI emerged as a common and severe complication, affecting 34.0% of our psittacosis cohort—a rate markedly higher than that observed in typical CAP and previously underreported. The absence of a correlation between AKI and conventional inflammatory markers such as CRP and PCT, as well as isolated liver injury, suggests that renal impairment in psittacosis may not be solely attributable to the overall inflammatory burden or direct hepatic dysfunction. Rather, our analysis indicates that mechanisms involving systemic tissue hypoxia/injury and metabolic stress may play a significant role.

In this study, we introduce a novel perspective by identifying the BAR and LDH as significant independent correlates and predictors of AKI. The BAR is particularly noteworthy due to its dual capacity to reflect renal perfusion stress—via BUN, which is sensitive to hypoperfusion and catabolism—and systemic nutritional-inflammatory status through albumin levels (Huang et al., 2020; Shi et al., 2024). In the context of psittacosis, factors such as high fever, reduced intake, and hypermetabolism likely contribute to elevated BUN levels, while intense inflammation leads to albumin depletion, collectively increasing the BAR. Its superior predictive performance, with the AUC of 0.88, surpasses that of LDH and traditional markers, underscoring its clinical utility as an integrative and readily accessible bedside index. Similarly, elevated LDH levels, a non-specific marker of cellular necrosis and tissue hypoxia, were correlated with AKI, reinforcing the role of global ischemic or cytotoxic injury in the pathogenesis of psittacosis-associated renal damage (Henry et al., 2020). The combined use of BAR and LDH enhances predictive accuracy, suggesting that they capture complementary pathways—namely, metabolic stress and cellular injury—that contribute to the development of AKI.

The distinct disease trajectory of psittacosis is characterized by a shorter duration from onset to hospital admission, followed by an extended period of hospitalization. This rapid progression is likely indicative of the high virulence of the pathogen and an intense host immune response, which contribute to early clinical deterioration. The extended hospitalization duration may be partially due to delays in administering appropriate antimicrobial therapy, as initial empirical treatments for CAP often lack efficacy against intracellular pathogens such as Chlamydia psittaci (Rello and Sabater-Riera, 2022). The subsequent swift clinical and biochemical improvement observed upon transitioning to omadacycline highlights the critical importance of early and accurate etiological diagnosis in shortening the disease course. Additionally, the development of AKI independently contributed to prolonged hospital stays, underscoring its significant impact on increasing healthcare resource utilization.

### 4.1 Limitations and future directions

This study is subject to limitations characteristic of its retrospective, single-center design. Although the sample size is considerable for a psittacosis cohort, it restricts the capacity for subgroup analyses. The absence of data on fluid balance, comprehensive medication histories (particularly concerning nephrotoxic drugs), and sequential biomarker measurements may introduce confounding factors into the analysis of AKI. To substantiate our findings, clarify the temporal relationship between inflammation and AKI, and investigate the potential direct renal tropism of Chlamydia psittaci, future research should involve prospective, multi-center studies incorporating serial biomarker sampling, including biomarkers such as BAR, LDH, and novel kidney injury molecules.

## 5 Conclusion

In summary, psittacosis pneumonia manifests as a systemic immuno-inflammatory syndrome with a significant risk of multi-organ damage, particularly AKI. Traditional inflammatory markers do not indicate the risk of AKI; however, it is effectively predicted by the composite index BAR and the cellular injury marker LDH. These findings support the early calculation of the BAR index in cases of suspected or confirmed psittacosis to identify patients at elevated risk for AKI and extended hospitalization, thereby facilitating closer monitoring and timely supportive interventions. Recognizing this distinct clinical profile is essential for clinicians to enhance diagnostic accuracy, anticipate complications, and optimize the management of this re-emerging zoonotic disease.

## Data Availability

The original contributions presented in the study are included in the article/supplementary material. Further inquiries can be directed to the corresponding authors.
